# Working with community health workers to improve maternal and newborn health outcomes: implementation and scale-up lessons from eastern Uganda

**DOI:** 10.1080/16549716.2017.1345495

**Published:** 2017-08-29

**Authors:** Gertrude Namazzi, Monica Okuga, Moses Tetui, Rornald Muhumuza Kananura, Ayub Kakaire, Sarah Namutamba, Aloysius Mutebi, Suzanne Namusoke Kiwanuka, Elizabeth Ekirapa-Kiracho, Peter Waiswa

**Affiliations:** ^a^ Makerere University, School of Public Health (MakSPH), Kampala, Uganda; ^b^ Centre of Excellence for Maternal and Newborn Health Research, Makerere University, Kampala, Uganda; ^c^ Umeå International School of Public Health (UISPH), Umeå University, Umeå, Sweden; ^d^ Global Health Division, Karolinska Institutet, Stockholm, Sweden

**Keywords:** MANIFEST - Maternal and Neonatal Implementation for Equitable Systems Study, Maternal and newborn health, community mobilization, community health workers, implementation science, training and supervision

## Abstract

**Background**: Preventable maternal and newborn deaths can be averted through simple evidence-based interventions, such as the use of community health workers (CHWs), also known in Uganda as village health teams. However, the CHW strategy faces implementation challenges regarding training packages, supervision, and motivation.

**Objectives**: This paper explores knowledge levels of CHWs, describes the coverage of home visits, and shares lessons learnt from setting up and implementing the CHW strategy.

**Methods**: The CHWs were trained to conduct four home visits: two during pregnancy and two after delivery. The aim of the visits was to promote birth preparedness and utilization of maternal and newborn health (MNH) services. Mixed methods of data collection were employed. Quantitative data were analyzed using Stata version 13.0 to determine the level and predictors of CHW knowledge of MNH. Qualitative data from 10 key informants and 15 CHW interviews were thematically analyzed to assess the implementation experiences.

**Results**: CHWs’ knowledge of MNH improved from 41.3% to 77.4% after training, and to 79.9% 1 year post-training. However, knowledge of newborn danger signs declined from 85.5% after training to 58.9% 1 year later. The main predictors of CHW knowledge were age (≥ 35 years) and post-primary level of education. The level of coverage of at least one CHW visit to pregnant and newly delivered mothers was 57.3%. Notably, CHW reports complemented the facility-based health information. CHWs formed associations, which improved teamwork, reporting, and general performance, and thus maintained low dropout rates at 3.6%. Challenges included dissatisfaction with the quarterly transport refund of 6 USD and lack of means of transportation such as bicycles.

**Conclusions**: CHWs are an important resource in community-based health information and improving demand for MNH services. However, the CHW training and supervision models require strengthening for improved performance. Local solutions regarding CHW motivation are necessary for sustainability.

## Background

Globally, maternal and newborn mortality contribute greatly to the burden of preventable deaths, most of which occur in sub-Saharan Africa. Despite substantial progress and a decline in global maternal and child mortality by 44% and 53%, respectively, the targeted 75% and 66% drops in the Millennium Development Goals (MDGs) were not achieved [1]. Building upon the MDGs, the even more ambitious Sustainable Development Goals (SDGs), which call for major reductions in maternal, neonatal, and child mortality, and universal access to sexual and reproductive health services by 2030, were developed. These ambitious goals aim to reduce the maternal mortality ratio to less than 70 per 100,000 live births, end all preventable newborn deaths, and reduce neonatal mortality and still births to less than 12 per 1000 births by 2030 [1]. In Uganda, maternal mortality has stagnated at 438 deaths per 100,000 live births, with most mothers dying from postpartum hemorrhage, whereas neonatal mortality stands at 27 per 1000 live births. While 95% of women attend antenatal care at least once, only 57% of births occur in health facilities, and just 59% of births are attended by a skilled attendant [2].

Preventable maternal and newborn deaths can be averted through simple evidence-based interventions, such as the use of community health workers (CHWs) – an intervention that has been tried in many settings [3–5]. In low- and middle-income countries, there is increasingly more engagement of CHWs in healthcare as they are critical in increasing universal access to healthcare provision [3,4,6–8]. Evidence from Asia suggests that in areas with limited or no access to facility-based care, CHWs have a substantial impact on neonatal mortality [9–12]. Although studies from Africa did not register an impact of CHWs on neonatal mortality, they showed improvement in community knowledge of maternal and newborn danger signs, birth preparedness, and newborn care practices [8,13–15]. This may be attributed to the variation in the CHW packages used across different contexts globally.

In general, CHWs receive a basic training recognized by the national health certification authority, usually the Ministry of Health [16]. The length of this training varies in different countries. In Uganda, training lasts for 5 days [17], while in other countries such as Malawi it lasts for 10 weeks [18], and it even lasts for up to 1 year in Ethiopia [19]. In addition, supplementary regular training, aimed at maintaining a good level of knowledge and skills, has been found to be essential [6,20,21]. CHWs are trained to conduct home visits to promote environmental hygiene and sanitation; offer health education; collect data on vital statistics; and treat uncomplicated malaria, respiratory infections, and diarrhea. They are also trained to help communities recognize maternal danger signs and newborn danger signs and facilitate referral of patients with these signs to the nearest health facilities for management [22,23]. In Bangladesh, CHWs also treat neonatal sepsis with injectable antibiotics [24].

In Uganda, CHWs, also known as village health teams (VHTs), are literate men or women chosen by the community, and trained to deal with the simple health problems of individuals within the community, and to work in close relationship with the health services [17]. The VHT strategy in Uganda has been in use since 2002, albeit with implementation challenges. While the CHW structure is a nationally accredited program, it has been implemented mainly by development partners. A nationwide scale-up program has been challenged by several factors. Central to these have been the costs of implementation, limited synthesized evidence on how to scale up, multiple competing fragmented partners, lack of recognition of CHWs’ contribution, and implementation challenges such as inadequate training packages, disease-specific training, supervision modalities, remuneration, and motivation [13,25,26]. For effective implementation and scale-up, there is a need for better alignment and integration of VHTs into government health systems, while retaining flexibility for programs to innovate and respond to local needs [26]. This paper explores the knowledge levels of the CHWs, describes the coverage of home visits, and shares lessons learnt from the setting up and implementation of the CHW strategy.

## Methods

This study was part of the MANIFEST study, which employed a quasi-experimental study design with intervention and comparison arms in the three districts of Kamuli, Pallisa, and Kibuku [27]. The estimated population in this area is 1,075,242, with an annual population growth rate of 3% [28]. The MANIFEST intervention was implemented in half of each district, while the other half served as the comparison area and received the routine care offered by the Ministry of Health of Uganda, with no additional training, supervision, and mentorship of CHWs and facility-based health providers. The intervention had two main components: community mobilization and sensitization, and the health provider and management capacity building for better quality services. The community mobilization and sensitization strategy involved the use of CHWs and radio talk shows. This paper focuses on the use of CHWs for improved maternal and newborn care.

### Study subjects

To undertake community mobilization and sensitization to stimulate demand for maternal and newborn health (MNH) services, two CHWs from each of the villages in the intervention arm were selected and trained. The CHWs were drawn from a pool of existing community volunteers, such as drug distributors and previously selected CHWs. They were then trained for a period of 5 days to prepare them to undertake home visits (two during pregnancy and two within the first week after delivery) and community dialogues on maternal and newborn issues. CHWs’ activities were supported and supervised by ‘super CHWs’ (peer supervisors) and trained health assistants and health workers through two strategies: directly observed supervision (DOS) during home visits, and combined meetings of CHWs, supervisors, and district health management teams (DHMTs). The overall supervision of the implementation in each district was done by the district health officer (DHO) and a study (MANIFEST) focal person (assistant DHO in charge of maternal and child health) (Text Box 1). A more comprehensive description of this intervention is given in the protocol paper [27].

**Text Box 1.** Community health workers (CHWs) intervention.

Selection of CHWs • Recruited community drug distributors in each village • 1680 CHWs selected: two per village Training of district trainers for CHWs • 4 day training of midwives, health assistants, clinical officers • 36 trainers trained: 12 per district Training of CHWs • 5 day training using the standard national training module • District and national trainers used Training of CHW supervisors • 2 day training • 79 supervisors trained **Supervision of CHWs** • Directly observed supervision (DOS) by health workers and health assistants • Monthly meetings of CHWs with super CHWs • Quarterly meetings of CHW with supervisors and district health management team

### Data collection

Data were collected using both quantitative and qualitative research methods of data collection. The study population comprised CHWs in the intervention area, health workers who played the supervisory role for the CHWs, community leaders and DHOs who were in charge of the overall supervision at district level, as well as women of reproductive age resident within the study area. To assess the knowledge levels of CHWs on maternal and newborn care, we conducted a pre- and post-test exercise during their training. In addition, we carried out a mini-survey of the same CHWs 1 year into the implementation to assess knowledge retention. Our sample size was informed by the Leslie Kish formula for cross-sectional studies [29]. Using the formula *n* = *Z* ^2^ *PQ*/*d* ^2^, we assumed a proportion of 50% for knowledge of danger signs by CHWs, and a design effect of 1, which gave us a sample size of 402 CHWs. We assumed a design effect of 1 since we never expected a cluster sampling effect. We also never anticipated for non-response, since all CHWs were required to take pre- and post-training tests before enrolment. The CHWs were subjected to the pre- and post-training test to identify gaps in their knowledge and address them appropriately during the training, and 1 year later, were again subjected to the same test to assess knowledge retention. The CHWs in the control area did not receive additional training from the study, hence were not subjected to a pre- and post-training test.

Because the training of CHWs was conducted concurrently across all three districts, care was taken to ensure that the 402 CHWs’ pre-/post-test results analyzed were drawn from all the districts. The sample size was drawn proportionately to the number of CHWs trained in each of the districts. These were conveniently obtained as we trained the CHWs, until the desired sample size was reached. However, 1 year into the implementation, more results of CHWs (*n* = 513) were drawn from the three districts and analyzed for knowledge retention.

To describe the coverage of CHW home visits, data on CHW coverage and source of maternal newborn health information were extracted from the project baseline and final surveys of 2293 and 1946 mothers, respectively, who had delivered 12 months before the survey [27]. The data collection during the baseline (September 2012) and final surveys (August 2015) were collected by trained and experienced research assistants from both the intervention and comparison areas of the three districts.

To explore lessons learnt from the CHW strategy, the authors reviewed quarterly CHW meeting minutes and reports from August 2013 to July 2015, where information on supervision, challenges, and motivation was captured. In addition, we interviewed 10 key informants: all three DHOs, three MANIFEST project focal persons, three health workers involved in CHW supervision (one per district), and one health assistant who also served as a CHW trainer. We also conducted 15 in-depth interviews: six with super CHWs (two per district) and nine with CHWs (three per district), of whom six were female, with an average age of 35 years. Respondents for these interviews were purposively sampled among those who were involved in the MANIFEST intervention. The respondents were asked to give their opinions on the effect of supervision on CHW performance, the challenges faced by CHWs, and what motivates CHWs to continue working as volunteers. Key informant and in-depth interview guides were pre-tested in an area outside the study area and validated. Interviews were conducted by researchers experienced in qualitative work and were audio recorded.

### Data analysis

Quantitative data were analyzed using STATA version 13.0 for descriptive statistics such as frequencies and percentages in terms of coverage and source of MNH information. CHW pre- and post-test results were analyzed for knowledge retention in terms of overall percentage scores, and specifically on maternal and newborn danger signs and practices. In both tests, CHWs were asked to mention four maternal danger signs during pregnancy and four after delivery, three newborn danger signs, and three essential newborn care practices. Additional questions were asked on thermal care, breastfeeding, and birth preparedness. Each correct question was given a maximum weight of 1 and the overall maximum total of each test was 23. CHWs’ pre-test, post-test, and 1 year test marks were then computed as percentages. We also used ordered logistic regression to ascertain the predictors of knowledge of maternal and newborn care among the CHWs, controlling for age, gender, religion, educational level, and duration of service as a CHW. CHWs were considered knowledgeable if they mentioned three danger signs for each category (pregnancy, postnatal, newborn) and three essential newborn care interventions. Ordered logistic regression was used because the study outcome was at least three ordered categories classified in their order of magnitude. In this study, we presented the results for the highest categories (mentioned all three danger signs) versus those who did not have any knowledge at all as the reference category. Survey data were analyzed for CHW coverage at baseline and the end of the project, and for the different sources of MNH information for mothers in the intervention and comparison districts.

The qualitative data were transcribed and analyzed using thematic analysis following the steps recommended by Braun and Clarke [30]. The first author read through all the transcripts and field notes from the key informant interviews and in-depth interviews multiple times to become familiar with the data. Data were coded and manually analyzed. The same codes were applied to the data (extracted by the second author) from the quarterly review meeting reports. The codes were grouped by checking for relationships and links between them to form sub-themes, which were later developed into themes through a similar process.

Key themes explored included lessons on selection of CHWs, supervision of CHWS, motivation of CHWs, and challenges faced by CHWs.

### Ethical approval and consent to participate

Ethical approval was obtained from Makerere University School of Public Health Higher Degrees Research and Ethics Committee, and Uganda National Council of Science and Technology. Permission was obtained from the district health offices in the three study districts. Written informed consent was obtained from all the study respondents before the interviews.

## Results

### Socio-demographic characteristics of CHWs under the MANIFEST intervention

Table 1 highlights the socio-demographic characteristics of the selected CHWs. Most (60%) of the CHWs were aged between 21 and 40 years, and had reached a secondary level of education. Almost all (96%) CHWs had previously worked as community volunteers for more than 5 years, with few (3.7%) having formal employment.

**Table 1.** Socio-demographic characteristics of community health workers (CHWs).

Characteristic (*N* = 402)	*n* (%)
Gender	
Female	217 (54.1)
Male	185 (45.9)
Age group (years)	
18–20	6 (1.5)
21–30	87 (21.6)
31–40	154 (38.3)
41–50	116 (28.9)
> 50	39 (9.7)
Educational level	
None	3 (0.7)
Primary	127 (31.5)
Secondary	244 (60.7)
Tertiary	29 (7.1)
Religion	
Catholic	100 (25.0)
Muslim	40 (9.9)
Protestant	202 (50.3)
Other	59 (14.8)
Marital status	
Married	356 (88.6)
Never married	12 (2.9)
Separated/widowed	34 (8.4)
Employment	
Formal employment	15 (3.7)
Peasant	270 (67.2)
Student	9 (2.3)
Unemployed	108 (26.8)
Period working as a CHW (years)	
< 1	6 (1.5)
1–5	10 (2.6)
> 5	386 (96.0)

### CHWs’ knowledge at training and retention level after 1 year of implementation

The CHWs’ knowledge of danger signs and essential home-based newborn care improved following the training, as indicated by the pre/post mean scores of 41.3% versus 77.4%, and 79.9% 1 year later. Before the training (pre-test), only 27.6% of the CHWs could mention three danger signs during pregnancy (Table 2). This improved to 89.3% and 81.9% in the post-test and 1 year later, respectively. Knowledge of three maternal danger signs after delivery and danger signs in the newborn baby was even lower in the pre-test, at 16.7% and 20.8%, respectively. Following training, these figures improved markedly to 73.9% and 85.5%, respectively. However, 1 year later, the knowledge of three newborn danger signs dropped to 58.9%, while that for maternal danger signs was maintained beyond 70%. A similar decline was observed in knowledge of essential care interventions in the home to protect the newborn, from 75.3% (post-test) to 50.3% 1 year later during the implementation. The predictors of CHWs’ knowledge (post-test) of maternal danger signs following logistic regression were age and level of education (Table 3). The older (> 35 years) CHWs were more likely than their younger counterparts to know three maternal danger signs during pregnancy and after delivery. The CHWs who had reached a post-primary level of education were more than twice as likely to know three maternal danger signs (*p* < 0.001) and newborn danger signs (*p* < 0.05). However, the period of working as a CHW did not have any effect on CHWs’ knowledge.

**Table 2.** Community health workers (CHWs’) knowledge of maternal and newborn danger signs and essential care interventions at home.

	Pre-test *n* (%)	Post-test *n* (%)		1 year of implementation *n* (%)	
Variable	402	402	*p*^a^	513	*p*^a^
Danger signs during pregnancy					
Could not mention any	58 (14.5)	1 (0.3)	< 0.001	9 (1.7)	< 0.001
Could mention one danger sign	113 (28.1)	10 (2.5)		26 (5.1)	
Could mention two danger signs	120 (29.9)	32 (8.0)		58 (11.3)	
Could mention three or more	111 (27.6)	359 (89.3)		420 (81.9)	
Maternal danger signs after birth					
Could not mention any	71 (17.7)	14 (3.5)	< 0.001	19 (3.7)	< 0.001
Could mention one danger sign	140 (34.9)	26 (6.5)		43 (8.4)	
Could mention two danger signs	123 (30.7)	57 (14.2)		88 (17.2)	
Could mention three danger signs	68 (16.9)	297 (73.9)		363 (70.8)	
Danger signs in newborns					
Could not mention any	89 (22.1)	3 (0.8)	< 0.001	29 (5.7)	< 0.001
Could mention one danger sign	125 (31.2)	19 (4.7)		60 (11.7)	
Could mention two danger signs	104 (25.9)	36 (9.0)		120 (23.4)	
Could mention three danger signs	84 (20.8)	344 (85.5)		302 (59.3)	
Essential care interventions in the home to protect the new baby					
Could not mention any	74 (18.3)	11 (2.7)	< 0.001	4 (0.8)	< 0.001
Could mention one	116 (28.9)	26 (6.5)		73 (12.2)	
Could mention two	152 (37.8)	62 (15.4)		129 (25.2)	
Could mention three	60 (14.9)	303 (75.3)		307 (59.8)	

^a ^Association determined using Pearson’s chi-squared test considering pre-test results as reference.

**Table 3.** Predictors of community health workers’ (CHWs’) knowledge (post-test) of maternal and newborn danger signs, and newborn essential care interventions, using ordered logistic regression.

	Model 1	Model 2	Model 3	Model 4
	Knowledge of 3 pregnancy danger signs	Knowledge of 3 postnatal danger signs	Knowledge of 3 newborn danger signs	Knowledge of 3 newborn essential care interventions
	Adj. OR (95% CI)	Adj. OR (95% CI)	Adj. OR (95% CI)	Adj. OR (95% CI)
Gender				
Male**^a^**	1.00	1.00	1.00	1.00
Female	0.79 (0.54,1.16)	0.82 (0.58,1.16)	0.87 (0.62,1.23)	1.10 (0.76,1.57)
Age group (years)				
18–24**^a^**	1.00	1.00	1.00	1.00
25–34	1.97 (0.90,4.33)	2.95** (1.36,6.39)	1.41 (0.66,3.03)	1.15 (0.52,2.53)
35–44	2.50* (1.11,5.64)	2.98** (1.35,6.59)	1.30 (0.60,2.83)	1.07 (0.48,2.39)
≥ 45	2.27* (1.00,5.12)	2.91** (1.31,6.46)	1.33 (0.61,2.93)	1.16 (0.52,2.62)
Educational level				
Primary**^a^**	1.00	1.00	1.00	1.00
Post-primary	2.03*** (1.39,2.97)	2.26*** (1.58,3.21)	1.55* (1.09,2.20)	1.55* (1.07,2.24)
Religion				
Catholic**^a^**	1.00	1.00	1.00	1.00
Protestant	0.88 (0.56,1.38)	1.50* (1.00,2.26)	1.10 (0.74,1.65)	1.06 (0.67,1.62)
Muslim	0.63 (0.32,1.21)	1.35 (0.73,2.48)	1.48 (0.81,2.74)	0.97 (0.51,1.84)
Other	0.65 (0.37,1.14)	1.33 (0.79,2.25)	0.97 (0.58,1.63)	1.03 (0.59,1.80)
Time working as VHT				
< 1 year**^a^**	1.00	1.00	1.00	1.00
≥ 1 year	1.09 (0.48,2.47)	1.61 (0.75,3.45)	0.80 (0.36,1.79)	0.90 (0.39,2.07)

**^a ^**Reference category.

Adj. OR, adjusted odds ratio; CI, confidence interval; VHT, village health team.

*p < 0.05, **p < 0.01, ***p < 0.001.

### Coverage of CHW home visits

CHWs’ coverage for at least one home visit to either pregnant or newly delivered mothers in the first week after delivery was 57.3%, while coverage of home visits after delivery was 37.5%. The drop in postnatal care coverage was due to challenges encountered in conducting home visits after delivery. This, in a way, also affected the newborn referral, which covered about 5% (1037) of the total deliveries registered by CHWs in the implementation period.

Most CHWs (10 out of 15) expressed difficulties in timely identification of a newly delivered mother given the short window of opportunity of 7 days when they were required to conduct the postnatal home visits. The CHWs pointed out that although attempts were made to capture the telephone numbers of the mothers during the antenatal home visits, CHWs still experienced limitations in contacting these mothers since some mothers lacked phones or the phones were not charged. The other option the CHWs used was to ask the mother to send a person to inform them of the birth. However, the quantitative results showed improvements in mothers’ knowledge of danger signs, birth preparedness, maternal health service utilization (institutional delivery), and newborn care practices such as putting nothing on the cord and antenatal care utilization for the four recommended times. For instance, early antenatal care attendance at the end of the project was observed more among women in the intervention area who were visited by the CHWs, and health facility deliveries increased from 65.2% to 75.3% in the intervention area.

In addition, the effect of home visits by the CHWs was reflected in the sources of information for mothers on danger signs and birth preparedness. The findings show that in the intervention area the main sources of MNH information for mothers were CHWs during home visits (83.9%) and community dialogue meetings (82.7%), followed by the radio (79.2%) and health facilities (60%), whereas in the comparison area, the main source of MNH information was the health facility, at 54.8%. Similarly, CHWs’ reports were also noted to be an important resource for the health management information system (HMIS) for pregnancy outcomes. CHWs made monthly reports on pregnancies identified and pregnancy outcomes. For instance, from January to September 2014, CHWs recorded 145 low-birth-weight babies and 145 newborn deaths, compared to 93 newborn deaths recorded in the facility-based HMIS for the same period.

### Lessons learnt from setting up and implementing the CHW strategy

#### Selection of CHWs

The CHWs selected were already working as community medicine distributors (CMDs); however, the national criteria had not been followed in selecting these CMDs. Consequently, some of the CHWs did not know how to read and write, and others were not ready to carry out the new roles of moving from household to household for home visits. The national selection criteria for CHWs require village meetings to be held to explain the job description and then choose the most suitable candidate, rather than taking on any person in the community who has previously been involved in some health activity.

#### Supervision of CHWs

Support supervision sessions were found to be helpful in reinforcing the knowledge and skills of CHWs in community-based maternal and newborn care, including referral to health facilities. We noted that CHWs require regular, frequent, and continuous support supervision for motivation and to improve performance. During the first year of the implementation period, CHWs received a total of 16 supervisory sessions, comprising 12 monthly directly observed support supervisions and four quarterly group meetings, to build their capacity to conduct quality home visits. Towards the end of the study, CHWs seemed more knowledgeable and confident of what they were supposed to do, and therefore we started tapering down the health workers’ supervisory sessions of DOS. However, this reduced supervision was noted to have negatively influenced the morale of CHWs in conducting home visits. Thus, under the guidance of the district health team, we later intensified the monthly peer-to-peer support through ‘super CHWs’, to replace the DOS sessions by health workers from the nearby facilities, who were noted to be overworked. The district-based key informants pointed out that the use of health workers to supervise CHWs from their catchment areas is important in building CHWs’ technical skills, as well as improving the community–facility links and relationships. However, they also highlighted the high workload for frontline health workers that may have hindered the adequacy and quality of this kind of supervision:

'Use of health workers to supervise CHWs is important, since they have the technical knowledge and skills, and support community–facility linkages. However you find that there is a shortage of health workers at health facilities due to study leave or some are off duty. And the CHW supervision work being an added assignment can create work overload and affect their performance at both the facilities and community level.' (MANIFEST focal person, Kibuku District)

#### Motivation of CHWs

In this study, CHWs had both monetary and non-monetary incentives. The latter included a T-shirt for identification, regular supervision, and provision of working materials, while the former was a transport refund of about 6 USD per quarter. On many occasions, CHWS asked for new bicycles or a fund to repair their old ones, which the project was unable to provide. It was generally observed that, much as the non-monetary incentives kept the CHWs’ motivation high and minimized the dropout rate (3.6%), the monetary incentives were equally important motivators, given the heavy load of community mobilization and sensitization. This was confirmed by expressions from the CHWs and their supervisors, as illustrated by the following quotations:

'Ok, we (CHWs) do a lot of work and at times you reach an extent and you hate yourself; that why did I join this program? Because today, one has got pregnant, then the other has delivered, and you have to conduct home visits for them all, yet the money is a little small. They give us 20.000/= after three months and if we have not done dialogue meetings, we don’t get the money (10.000/=) which is given after sensitizing them (community members in a dialogue); that is small money, because we are the ones who work; we are the ones playing the biggest role.' (Super CHW, Kamuli District)

'Yes that’s motivation; somehow somewhere they should be motivated once in a while. We are not saying that give them salary and they are motivated and those things to use, like a bicycle when it has not reached being broken down, may be in a quarter, and the uniforms they used to give them and those T-shirts also motivate them. They become very happy; recently they gave them these T-shirts for malaria and they are there and working with a lot of energy. When there is such motivation, they also go on doing what they should do.' (Health assistant, Kamuli District)

In addition, CHW associations were noted by the supervisors to enhance CHW motivation and hence performance. It was observed that where CHWs had an association, whether at parish or sub-county level, their reporting was better and they were unlikely to drop out. Such CHWs were more likely to work as a team and this also made the supervisory role of the super CHW easier. Consequently, CHWs were encouraged and supported to start up associations/saving groups using the experiences of those that were already well established.

#### Challenges faced by CHWs during implementation

CHWs complained of a lack of means of transport, such as bicycles, and a lack of protective items such as umbrellas and gumboots, especially during the rainy season. They also preferred the use of loudspeakers to mobilize community members to attend dialogue meetings, rather than moving from house to house. In addition, they complained of inadequate quality of care at health facilities to meet the increasing demand, which was expressed by the mothers whom they referred to health facilities. This discouraged them, to some degree, from referring clients to health facilities. CHWs also expressed the challenge of high expectations and demands on them, from the community they served and their supervisors, compared to their capabilities and level of motivation and incentives.

'We offer sensitization to pregnant and newly delivered mothers during home visits and through the village meetings. We talk about care for mothers and their babies, domestic hygiene, family planning, and good feeding. However, we face a challenge of them requesting things from us such as bed nets, medicines, maama kits, etc., which we do not have, since the project does not provide them. Maybe if there is no transport to hospital, there I can come in and offer my bicycle. I can also give her company to the health facility.' (VHT, Pallisa District)

Furthermore, CHWs faced challenges in bringing men on board to support their wives (pregnant and newly delivered mothers) in maternal and newborn care, especially at the beginning of the study. Passing on information to husbands was problematic, either because they were rarely found at home or because of perceptions that the information was designed for women. However, through the community dialogue meetings, more men were reached, and with time they started appreciating the messages. Men started giving funds to their wives to save for MNH care and emergencies. They also started buying birth items before delivery and escorting their wives to antenatal care at the health facilities. However, some men expressed frustration at not being involved by their own wives. Men complained that often their wives did not even inform them that they were pregnant, and they did not even get to know when labor set in:

'There are also problems with women; some women do not want to disclose to their husbands that they are pregnant or that labor has started. They prefer to share with their own relatives, like sisters or their own mothers. How can a man then help?' (CHW Kibuku District)

Lastly, being involved in multiple projects was also found to be challenging to the CHWs. This made it difficult to maximize their performance levels. Hence, the CHWs felt torn apart or overloaded. We observed that the quality of CHW work and reporting in situations where several community-based activities were undertaken by the same CHWs and coincided were compromised.

## Discussion

The findings of this study show that CHWs are an important resource for sensitizing communities and improving demand for MNH services at community level. CHWs collect vital community-based MNH data that can be used to complement the facility-based HMIS. However, CHW implementation is plagued by many challenges, ranging from training to supervision and motivation of this important workforce. Understanding the dynamics of implementation of CHWs is paramount in informing practice and scaling up CHW strategies [31].

From our findings, CHWs played a role in generating demand for and utilization of MNH services, despite the average levels of coverage [32]. This can be attributed to CHW activities of home visits and community sensitization on MNH, through village dialogue meetings, as well as referral of the high-risk cases to nearby facilities. This is triangulated by another finding from the study, that the most common source of MNH information at a community level was the CHWs, although other information sources existed, including the media and peers. These findings are similar to those from other low- and middle-income countries, especially in Asia and in other parts of Africa, where CHWs have played an important role in reducing newborn morbidity by providing home-based care and referrals, and improving community knowledge on danger signs, as well as newborn care practices [10,12,33].

Notably, the study findings show that a challenge remains in improving newborn care practices at community level. In spite of the changes in newborn care practices being statistically significant, the absolute changes registered were minimal. For example, delayed bathing of newborns after 24 h increased by only six percentage points [32]. This may be due not only to the inadequate knowledge retention of CHWs on the overall newborn package, including care practices and danger signs, and challenges faced in conducting postnatal care home visits, but also to the ingrained cultural practices within the intervention study area. This finding has implications not only for the type of newborn training undertaken by CHWs, but also, ultimately, for newborn survival. It is therefore necessary to rethink and redesign the newborn training, supervision components, and selection criteria regarding the level of education of CHWs.

In Uganda and elsewhere, CHWs collect and maintain village household registers containing a variety of health-related information, including vital statistics on pregnant women, births, and maternal and newborn deaths [8,18]. Often, statistics, especially community deaths and births, are underreported in the routine HMIS, which is facility based. CHWs’ reports therefore play an important role in complementing the health-facility based information system by providing these vital statistics and providing the actual picture. Hence, district health teams should endeavor to integrate the two forms of reporting to provide a more comprehensive HMIS. Even though there are other reliable data sources, such as the demographic surveys and national census, these are conducted after long time intervals of 5 and 10 years, respectively. Therefore, a comprehensive HMIS data system, through the incorporation of CHW reporting, would serve as an appropriate tool for planning, as these data are collected on a quarterly basis.

This study found that although training of CHWs is necessary to impart the required maternal and newborn knowledge, it is not enough to sustain it. This was exemplified by the depreciation in knowledge levels of danger signs and care practices for newborns during a 1 year post-training CHW knowledge retention analysis. Knowledge of danger signs and care practices among newborns was observed to be low, relative to the knowledge on maternal danger signs, the retention of which remained stable (> 70%). This could point to a gap in the training modules for the newborn component and a need to strengthen it during these training sessions and in the quarterly review meetings.

From our findings, the positive predictors of VHT knowledge for MNH were age and educational level. Surprisingly, although duration of service as a CHW could have influenced knowledge levels, it was not statistically significant. This has implications for policymakers and programmers. In Uganda, the current criteria for CHW selection do not emphasize the level of education, as long as the person knows how to read and write, but recommends those who have previously worked as CHWs [17]. The selection criteria require revision to incorporate post-primary level of education and older age groups. While several studies have shown that CHWs require regular training to maintain a good level of knowledge and skills [6,20,21], the MANIFEST intervention involved a 5 day training course, where a wide range of aspects was handled, and this could have compromised the quality. However, initial training was followed by short, regular sessions of integrated support supervision to reinforce knowledge and skills focusing on a few issues at a time, particularly in the CHWs’ first year of work.

In our study, we used a model of health workers complemented by peer supervisors to carry out support supervision of CHWs. This model is sustainable on many fronts in our setting of inadequate human resources for health and financial constraints. The use of peer support supervisors is certainly a relatively cheap and reliable model, and CHWs can relate to and learn from their peers. However, the peer supervisors require continued regular engagement to update their knowledge. This is in comparison with a retraining model, which has higher cost implications and is not sustainable. Some other studies contradict these findings; for example, in a Zambian study evaluating the performance of CHWs, support supervision did not influence their performance [34]. However, in Zambia, support supervision was irregular, and no standard method or checklist was used during supervisory visits.

The findings of the present study show that non-monetary incentives such as T-shirts, certificates, and *musawo* (doctor) status in the community can motivate CHWs to continue working. However, monetary incentives and means of transportation, such as bicycles, were found to be essential for CHWs to undertake their roles. These findings concur with results from many other studies conducted in Africa and Asia which show that giving CHWs incentives has a positive impact on their performance [21,23,35,36]. A study in South Africa attributed the high rates of CHW attrition to a lack of monetary incentives [37]. However, although monetary incentives may increase productivity and lower CHW attrition rates, they could inadvertently lead to a dependency syndrome, whereby the productivity outputs are tied to the funds. Furthermore, these monetary incentives may not be sustainable. This has implications for Uganda, which is planning to adopt a CHW model similar to that used in Ethiopia [22]. We will require a thorough costing of such a model to ensure sufficient funds for its success. Non-monetary incentives are more sustainable. Studies in Bangladesh and India have shown that community support and respect are key non-monetary incentives for CHWs [38,39]. Similarly, in Columbia, CHWs ranked having influence in the community as the most important non-financial motivator of performance [40]. Given our study findings and the previous work, there is a need for both monetary and non-monetary incentives, as well as some transport facilitation to improve coverage and reporting rates.

### Study limitations and strengths

This study has some limitations. The sampling frame does not represent the diverse socio-cultural differences present in Uganda. Therefore, generalizability of the findings to a wider population may be restricted in some settings. In addition, the learning effects from the test and retest may have influenced the validity of knowledge results. Furthermore, there is no linkage of pre- and post-training and 1 year data at an individual level (panel data). Thus, the CHWs’ knowledge change 1 year later may be inconsequential. Despite these limitations, our study provides important insights into the implementation of the CHW strategy at a district level. Specifically, the findings add to the general body of knowledge on lessons for training, supervision, and motivation of CHWs for improved community mobilization in utilization of MNH services. These findings have wide implications for the implementation and scale-up of the CHW strategy.

## Conclusion

Through a participatory process with continuous engagement, training, and improvements in support supervision and links to functional health facilities, CHWs have the potential to become an important resource for sensitizing communities, improving demand for MNH services, as well as a source of community-based MNH information. Community interventions for newborn survival require optimal population coverage by CHWs, with context-specific selection, training, and remuneration. Therefore, to achieve better outcomes, the training and supervision models for the newborn component need to be strengthened. Furthermore, local solutions are necessary to ensure the sustainability of the CHW strategy, especially with regard to their motivation.

## Paper context

Many studies have investigated the use of CHWs, including the role of CHWs in improving utilization of maternal and newborn health (MNH) services, as well as training and supervision modalities of CHWs. This paper adds to this general body of knowledge on the use of CHWs in low-resource settings. In addition, it provides some predictors of levels of knowledge of the CHWs which have implications for the selection criteria. It also highlights strategies to address some implementation challenges, particularly supervision critical for improved performance and motivation, and illuminates the use of CHWs as a source of community-based MNH information complementary to facility-based information.
